# *ERCC2*, *ERCC1 *polymorphisms and haplotypes, cooking oil fume and lung adenocarcinoma risk in Chinese non-smoking females

**DOI:** 10.1186/1756-9966-28-153

**Published:** 2009-12-14

**Authors:** Zhihua Yin, Meng Su, Xuelian Li, Mingchuan Li, Rui Ma, Qincheng He, Baosen Zhou

**Affiliations:** 1 Department of Epidemiology, School of Public Health, China Medical University, Shenyang 110001, PR China; 2 Key Laboratory of Cancer Etiology and Intervention, University of Liaoning Province, Shenyang 110001, PR China; 3 Department of Internal Medicine, Liaoning Cancer Hospital & Institute, Shenyang 110042, PR China

## Abstract

**Background:**

Excision repair cross-complementing group 1 (ERCC1) and group 2 (ERCC2) proteins play important roles in the repair of DNA damage and adducts. Single nucleotide polymorphisms (SNPs) of DNA repair genes are suspected to influence the risk of lung cancer. This study aimed to investigate the association between the *ERCC2 *751, 312 and *ERCC1 *118 polymorphisms and the risk of lung adenocarcinoma in Chinese non-smoking females.

**Methods:**

A hospital-based case-control study of 285 patients and 285 matched controls was conducted. Information concerning demographic and risk factors was obtained for each case and control by a trained interviewer. After informed consent was obtained, each person donated 10 ml blood for biomarker testing. Three polymorphisms were determined by polymerase chain reaction-restriction fragment length polymorphism (PCR-RFLP) method.

**Results:**

This study showed that the individuals with the combined *ERCC2 *751AC/CC genotypes were at an increased risk for lung adenocarcinoma compared with those carrying the AA genotype [adjusted odds ratios (OR) 1.64, 95% confidence interval (CI) 1.06-2.52]. The stratified analysis suggested that increased risk associated with *ERCC2 *751 variant genotypes (AC/CC) was more pronounced in individuals without exposure to cooking oil fume (OR 1.98, 95%CI 1.18-3.32) and those without exposure to fuel smoke (OR 2.47, 95%CI 1.46-4.18). Haplotype analysis showed that the A-G-T and C-G-C haplotypes were associated with increased risk of lung adenocarcinoma among non-smoking females (ORs were 1.43 and 2.28, 95%CIs were 1.07-1.91 and 1.34-3.89, respectively).

**Conclusion:**

*ERCC2 *751 polymorphism may be a genetic risk modifier for lung adenocarcinoma in non-smoking females in China.

## Background

In China, lung cancer is currently the most common cancer among men and the second one among women and is the leading cause of cancer-related deaths for both genders. The incidence and mortality rate of lung cancer in China urban populations have reached the number one among malignant tumors. Although the incidence and death rate of lung cancer is now declining in men, the incidence and death rate in women continues to increase. So in this sense it is more important to study the impact factors of lung cancer in female population. Adenocarcinoma accounts for about 40% of all lung cancer, with a higher incidence in women. It is the most frequent subtype occurring in those who have never smoked. The epidemiologic characteristics and risk factors of lung cancer in nonsmokers are not clear. As we know, many lung cancer patients didn't have the history of smoking and a lot of smokers didn't develop lung cancer [1], suggesting that host susceptibility factors may play an important role in this disease.

Recent genetic susceptibility studies of cancer have focused on single nucleotide polymorphisms (SNPs) in candidate genes, among which DNA repair genes are increasingly studied because of their critical role in maintaining genome integrity. Excision repair cross-complimentary group 1 and group 2 (*ERCC1 *and *ERCC2*) are the important DNA repair genes, playing critical roles in nucleotide excision repair (NER) pathway which is the most important system to repair a wide variety of structurally DNA lesions, including bulky adducts, cross-links [2], oxidative DNA damage, thymidine dimmers [3]and alkylating damage [4]. The two genes are all located in chromosome 19q13.2-13.3. *ERCC2 *codes for an evolutionarily conserved helicase, a subunit of TFIIH complex which is essential for transcription and NER. ERCC1 protein is responsible for recognition of DNA damage and removal of the damaged nucleotides in NER. SNPs in exons of DNA repair genes may influence their protein activity, resulting in differences of individual NER and DNA repair capacity (DRC) that may affect the susceptibility of lung cancer. So we selected the common SNPs in exons of *ERCC2 *and *ERCC1 *gene and with the frequency of heterozygosity >5% in the present study. The common polymorphism of *ERCC1 *gene is at codon 118 (C > T substitution at exon 4, without amino acid change--Asn/Asn, rs11615). The common polymorphisms of *ERCC2 *gene is at codon 751 (A > C substitution at nucleotide position 35931, exon 23, Lys>Gln, rs13181) and codon 312 (G >A substitution at position 23951, exon 10, Asp>Asn, rs1799793). The polymorphisms at codon 312 and 751 have been studied extensively for their potential implication in cancer risk.

The effect of the *ERCC2 *and *ERCC1 *polymorphisms, and also of the haplotypes encompassing these two genes, on susceptibility of lung adenocarcinoma in non-smoking females has not been reported so far. In the present study, we describe a case-control study of lung adenocarcinoma in non-smoking female population in Shenyang, China, to evaluate the roles of the SNPs in *ERCC1 *and *ERCC2 *gene on risk of lung adenocarcinoma and explore the interaction between genetic polymorphism and exposure to environmental risk factors in the development of lung adenocarcinoma.

## Methods

### Study subjects and data collection

In this hospital-based case-control study, the case group consisted of 285 diagnosed nonsmoking female patients (between January 2002 and November 2007) with histologically confirmed lung adenocarcinoma. At the same time controls were selected from cancer-free patients with other lung diseases but free of cancer history and symptom. Controls were all non-smoking females and frequency matched to cases on age (± 5 years). Controls suffered mainly from bronchitis, pneumonias, fibrosis, sarcoidosis, chronic obstructive pulmonary disease and emphysema. The human investigations were approved by the Institutional Review Board of China Medical University, and informed consent was obtained from each participant or each participant's representatives if direct consent could not be obtained. All patients were all unrelated ethnic Han Chinese.

Each participant donated 10 ml venous blood and was interviewed to collect demographic data and environmental exposures at the time they were admitted to the hospital. Information concerning demographic characteristics, passive smoking, cooking oil fume exposure, fuel smoke exposure, family history of cancer, occupational exposure and dietary habit was obtained for each case and control by trained interviewers. Individual with a total of 100 cigarettes in his lifetime was defined as a smoker, otherwise he was considered as a non-smoker. For cooking oil fume exposure, participants were asked about the frequency of cooking and types of oils. Subjects were also asked "How often did the air in your kitchen become filled with oily 'smoke' during cooking?" For each of these questions, there were four possible responses ranging from "never", "seldom", "sometimes", to "frequently". Exposure for cooking oil fume was categorized as an indicator variable equal to 1 if participants reported frequently or sometimes, and equal to 0 otherwise.

### DNA isolation and genotyping

Genomic DNA samples were isolated by guanidine hydrochloride (GuHCl) method. SNPs were analyzed by polymerase chain reaction-restriction fragment length polymorphism (PCR-RFLP) method as described previously [5]. The PCR primers (Takara Biotechnology Dalian Co. Ltd., China) for amplifying DNA fragment containing the *ERCC2 *751Lys/Gln, 312Asp/Asn, and *ERCC1 *118Asn/Asn were 751 F5'-GCC CGC TCT GGA TTA TAC G-3' and R5'-CTA TCA TCT CCT GGC CCC C-3', 312 F5'-CTG TTG GTG GGT GCC CGT ATC TGT TGG TCT-3' and R5'-TAA TAT CGG GGC TCA CCC TGC AGC ACT TCC T-3', 118 F5'-AGG ACC ACA GGA CAC GCA GA-3' and R5'-CAT AGA ACA GTC CAG AAC AC-3', respectively. The PCR products were digested with restriction enzyme (New England Biolabs, Beverly, MA) PstI (for Lys751Gln), StyI (for Asp312Asn), and BsrdI (for Asn118Asn) to determine the genotypes. A 10% masked, random sample of patients was tested twice by different persons, and the results were found to be concordant for all of the masked duplicate sets.

### Statistical analysis

All statistical analyses were performed with Statistical Product and Service Solutions (SPSS) v13.0, if not otherwise specified. All of the tests were two-sided, and statistical significance was defined as P < 0.05. Pearson's chi-square test was used to compare the distribution of the demographic variables and examine differences in risk factors and genotypes, alleles and haplotypes between cases and controls. Hardy-Weinberg equilibrium (HWE) of the genotypes was tested by performing a goodness-of-fit χ^2 ^test. Unconditional logistic regression analysis was performed to calculate the odds ratios (ORs) with 95% confidence intervals (CIs) for estimating the association between certain genotypes and lung cancer. The stratified analyses and gene-environment interaction were evaluated by logistic regression models. On the basis of the observed frequencies of three SNPs, we used the SHEsis analysis platform to calculate linkage disequilibrium index (D' and r^2^) and infer haplotype frequencies [6,7].

## Results

Selected demographic variables and environmental risk factors for the 285 patients and 285 controls were listed in Table 1. All subjects were females and all cases were lung adenocarcinoma patients. Mean ages of cases and controls (mean ± S.D.) were almost identical (53.9 ± 12.0 and 54.1 ± 9.1 years, respectively). There were no significant differences in the distribution of family history of cancer, passive smoking, fuel smoke exposure, occupational exposures, and dietary habits between cases and controls. However the cases were more likely than the controls to report cooking oil fume exposure (OR 1.61, 95%CI 1.13-2.30, P = 0.009).

**Table 1:** Selected variable in cases and controls

Variable	Cases n (%)	Controls n (%)	P value
Female	285	285	
Age (years)	53.9 ± 12.0	54.1 ± 9.1	0.750
Income(yuan/month)	619.34 ± 374.59	557.11 ± 390.61	0.071
Education			0.779
Never	27 (9.5)	26 (9.1)	
Elementary school	133 (46.7)	145 (50.9)	
Junior school	85 (29.8)	76 (26.7)	
Senior school and upwards	40 (14.0)	38 (13.3)	
Family history of cancer	39 (13.7)	27 (9.5)	0.116
Passive smoking	174 (61.1)	162 (56.8)	0.307
Fuel smoke exposure	84 (29.5)	78 (27.4)	0.577
Cooking oil fume exposure	104 (36.5)	75 (26.3)	0.009

Table 2 presents the distribution of *ERCC2 *751, 312 and *ERCC1 *118 polymorphisms in cases and controls. The frequencies of the 751C, 312A and 118T allele in the controls were 0.08, 0.05 and 0.21, respectively. All allele distributions were consistent with Hardy-Weinberg equilibrium. Among these SNPs, heterozygous carriers of the *ERCC2 *751AC genotype had a 1.66-fold risk of lung adenocarcinoma compared with those carrying the homozygous wild genotype (95%CI 1.07-2.59, P = 0.024). Individuals carrying *ERCC1 *118TT homozygote genotype had a 2.01-fold (marginally significant) increased risk of cancer compared with the wild genotype (95%CI 0.99-4.10, P = 0.054). *ERCC2 *312 polymorphism was not associated with risk of lung adenocarcinoma in this study. Considering the problem of sample size, further analyses were carried on by combining the heterozygous variant genotype with the homozygous variant genotype in three polymorphisms. As a result, the combined *ERCC2 *751AC/CC was associated with an increased risk of lung adenocarcinoma with an adjusted OR of 1.64 (95%CI 1.06-2.52, P = 0.025).

**Table 2:** *ERCC2 *751, 312 and *ERCC1 *188 polymorphisms and lung adenocarcinoma risk

Genotype	Cases n (%)	Controls n (%)	OR [95%CI]^a^	P value
*ERCC2 *751				
AA	220 (77.2)	242 (84.9)	1.00	--
AC	61 (21.4)	40 (14.0)	1.66 [1.07-2.59]	0.024
CC	4 (1.4)	3 (1.1)	1.28 [0.28-5.86]	0.751
AC/CC^b^	65 (22.8)	43 (15.1)	1.64 [1.06-2.52]	0.025
				
*ERCC2 *312				
GG	246 (86.3)	255 (89.5)	1.00	--
GA	38 (13.3)	30 (10.5)	1.30 [0.78-2.17]	0.317
AA	1 (0.4)	0 (0.0)	--^e^	--
GA/AA^c^	39 (13.7)	30 (10.5)	1.33 [0.80-2.21]	0.275
				
*ERCC1 *118				
CC	156 (54.7)	176 (61.8)	1.00	--
CT	104 (36.5)	96 (33.6)	1.19 [0.84-1.70]	0.334
TT	25 (8.8)	13 (4.6)	2.01 [0.99-4.10]	0.054
CT/TT^d^	129 (45.3)	109 (38.2)	1.29 [0.92-1.81]	0.139

Abbreviation: OR, odds ratio; CI, confidence interval.

^a^ORs were calculated by unconditional logistic regression and adjusted for age and cooking oil fume.

^b^OR and P value were calculated compared with wild genotype(AA) of *ERCC2 *751 polymorphism.

^c^OR and P value were calculated compared with wild genotype(GG) of *ERCC2 *312 polymorphism.

^d^OR and P value were calculated compared with wild genotype(CC) of *ERCC1 *118 polymorphism.

^e^OR and P value for this genotype could not be calculated.

In the stratified analyses, we found that the increased risk associated with *ERCC2 *751 variant genotypes (AC/CC) was more pronounced in individuals without exposure to cooking oil fume (OR 1.98, 95%CI 1.18-3.32, P = 0.010) and those without exposure to fuel smoke (OR 2.47, 95%CI 1.46-4.18, P = 0.001) (Table 3). Stratified by other environmental exposures, no statistically significant relationships were suggested (data not shown). We evaluated the interaction of genetic polymorphism with cooking oil fume exposure on lung adenocarcinoma using a logistic regression model. However, no evidence of significant gene-environment interaction was found (data not shown).

**Table 3:** *ERCC2 *751 SNP in relation to risk of lung adenocarcinoma, stratified by environmental exposures

Group	Cases n (%)	Controls n (%)	OR [95%CI]*	P value
Cooking oil fume exposure				
Non exposure				
*ERCC2 *751 AA	137 (75.7)	181 (86.2)	1.00	--
*ERCC2 *751 AC/CC	44 (24.3)	29 (13.8)	1.98 [1.18-3.32]	0.010
				
Exposure				
*ERCC2 *751 AA	83 (79.8)	61 (81.3)	1.00	--
*ERCC2 *751 AC/CC	21 (20.2)	14 (18.7)	1.03 [0.48-2.21]	0.940
				
Fuel smoke exposure				
Non exposure				
*ERCC2 *751 AA	150 (74.6)	182 (87.9)	1.00	--
*ERCC2 *751 AC/CC	51 (25.4)	25 (12.1)	2.47 [1.46-4.18]	0.001
				
Exposure				
*ERCC2 *751 AA	70 (83.3)	60 (76.9)	1.00	--
*ERCC2 *751 AC/CC	14 (16.7)	18 (23.1)	0.65 [0.30-1.41]	0.270

Abbreviation: OR, odds ratio; CI, confidence interval.

*ORs and 95%CIs were calculated by logistic regression, with the *ERCC2 *751 wild genotype (AA) as the reference group. ORs were adjusted for age.

We analyzed haplotypes using SHEsis program platform (Table 4). The three SNPs were in linkage disequilibrium in this study population. The haplotypes were composed of 3 coding SNPs (cSNPs) that locate across 68.734 kb on 19q13.3 region. Of 8 possible haplotypes, only 3 had frequencies of > 0.03 among both cases and controls and were included in the haplotype analysis. Three possible haplotypes represented 91.7% of the chromosomes for the cases and 94.0% for the controls. There was a statistically significant difference in the overall haplotype distribution between cases and controls (global test P < 0.001). According to our prior hypothesis and the SNP-based analyses, we considered the individuals with 751A-312G-118C haplotype to be the reference group for OR estimations. The A-G-T and C-G-C haplotypes were associated with increased risk of lung adenocarcinoma (ORs were 1.43 and 2.28, 95%CIs were 1.07-1.91 and 1.34-3.89, respectively). Patients without exposure to cooking oil fume were more likely to have the A-G-T and C-G-C haplotypes than did controls with ORs of 1.45 (95%CI 1.01-2.07) and 2.72 (95%CI 1.43-5.17), respectively. Among individuals with exposure to cooking oil fume, cases tended to be more likely to have the A-G-T and C-G-C haplotypes, however the findings were not statistically significant.

**Table 4:** Haplotype frequencies in cases and controls stratified by cooking oil fume exposure status

Haplotype	All subjects			Non exposure to cooking oil fume			Exposure to cooking oil fume		
			
	Cases (%)	Controls (%)	OR [95%CI]	Cases (%)	Controls (%)	OR [95%CI]	Cases (%)	Controls (%)	OR [95%CI]
A-G-C	348 (61.1)	406 (71.2)	1.00	226 (62.6)	307 (73.1)	1.00	119 (57.4)	98 (65.5)	1.00
A-G-T	132 (23.1)	108 (18.9)	1.43 [1.07-1.91]	80 (22.0)	75 (17.8)	1.45 [1.01-2.07]	55 (26.2)	34 (22.3)	1.33 [0.81-2.21]
C-G-C	43 (7.5)	22 (3.9)	2.28 [1.34-3.89]	30 (8.2)	15 (3.6)	2.72 [1.43-5.17]	14 (6.9)	8 (5.2)	1.44 [0.58-3.58]
P value			< 0.001			< 0.001			0.186

Abbreviation: OR, odds ratio; CI, confidence interval.

## Discussion

In recent years, the etiological study of lung cancer remains popular all over the world. But the results are inconsistent, and as we know besides tobacco smoking, other impact factors of lung cancer are not definitive. Cigarette smoking cannot fully explain the epidemiologic characteristics of lung cancer in Chinese women, who smoke rarely but have lung cancer relatively often. Undoubtedly non-smoking females are the ideal subjects to examine unknown, yet important environmental and genetic factors of lung cancer. And because adenocarcinoma represents the main subgroup of female lung cancer and is the most frequent subtype occurring in those who have never smoked, we studied the associations between risk for development of lung adenocarcinoma and multiple genetic polymorphisms in NER genes, as well as environmental factors among Chinese non-smoking female population.

Our previous studies found the significant associations between cooking oil fume exposure and lung cancer risk in Chinese non-smoking females [5,8]. The similar results were suggested in the present study. Individuals with exposure to cooking oil fume had a 1.61-fold increased risk of developing lung adenocarcinoma (P = 0.009). It was reported that cooking oil fume condensates can induce DNA damage [9] and result in the increase of DNA cross-links in a certain concentration [10]. Some study showed that exposure to cooking oil fume could inhibit cell growth, increase TGFbeta1 secretion, and induce oxidative stress in lung epithelial cells [11]. There are other studies suggesting the important roles of cooking oil fume exposure in lung cancer risk among nonsmoking women [12-14].

Based on our results, 751C variant allele of *ERCC2 *gene may contribute to risk of lung adenocarcinoma, whereas the *ERCC2 *312 and *ERCC1 *118 polymorphisms have no significant associations with lung adenocarcinoma among non-smoking females. The three SNPs selected in this study are all in exons of NER genes, which were considered to influence the protein activity, then decrease or increase the DNA repair capacity and finally associate with risk of cancer. The SNP at amino acid 751 of *ERCC2 *may be important in terms of ERCC2 protein activity [15], because it locate in the interactive domain, i.e. its helicase activator, p44, inside the TFIIH complex which is essential for transcription and NER [16]. The *ERCC2 *751 polymorphism was associated with higher levels of chromatic aberrations [17] and DNA adducts levels in non-smokers [18]. It was reported that *ERCC2 *751AC/CC was significantly defective in NER [19] and had a modulating effect on DRC [20]. These results suggested that *ERCC2 *751 polymorphism could result in a defect in NER and deficient DRC that may be responsible for increased susceptibility of cancer. Our results show that non-smoking females carrying *ERCC2 *751C variant allele were at an increased risk for lung adenocarcinoma compared with those carrying AA genotype (adjusted OR = 1.64). It suggests that the polymorphism of *ERCC2 *codon 751 plays an important role in the development of lung adenocarcinoma in non-smoking females. Previous studies in whole population got the same results [21,22]. However some reports found the non-correlation between the polymorphisms of *ERCC2 *gene and risk of lung cancer [23] or the opposite results [24]. The reason for these different conclusions is not clear now. Probably the size of the study population, the particularity of exposure to carcinogen in different populations and genetic differences of study subjects play important roles in it. In addition, the *ERCC2 *751 polymorphism may function through haplotype but not through genetic exon to affect protein activity. Is this appropriate for other SNPs in *ERCC2 *and *ERCC1*? The exact effects and mechanisms of these polymorphisms on lung cancer need further studies to elucidate.

As reported in previous study [25], the *ERCC2 *751C, 312A and *ERCC1 *118T alleles have been found to be in linkage disequilibrium. The exploratory haplotype analyses in the present study revealed the associations between the 751A-312G-118T and 751C-312G-118C haplotypes and the increased risk of lung adenocarcinoma in non-smoking females. The result showed that none of the analyzed haplotypes included the variant allele of *ERCC2 *312 polymorphism. Some European studies found that *ERCC2 *312 and 751 polymorphisms are closely linked and their effects are difficult to be separated. Our study indicated that *ERCC2 *751 polymorphism may indeed be of functional importance for lung adenocarcinoma among non-smoking female population. Because it is just a statistical estimation, further studies are required to confirm its biological validity.

Few molecular epidemiological studies of lung adenocarcinoma have been conducted thus far. This study is one of the largest studies among non-smoking female population to evaluate the correlation between NER gene polymorphisms and risk of lung adenocarcinoma, and also the gene-environment interaction in the development of lung adenocarcinoma. The strength of this study is its low rate of misclassification of outcome, as all of the cases were pathologically confirmed.

In summary, our study sheds light on the relationship between polymorphisms in the DNA repair gene *ERCC2 *and *ERCC1 *with environmental risk factors and susceptibility to lung adenocarcinoma in nonsmoking females in northeast China. Our results show that the *ERCC2 *751C allele or the haplotypes encompassing the variant allele are associated with risk of lung adenocarcinoma in Chinese nonsmoking female population. While the functional interpretation remains elusive, additional larger studies are needed to validate our findings.

## Conclusion

The results of the present study indicate that *ERCC2 *751 polymorphism (rs13181) might be a genetic risk modifier for lung adenocarcinoma in non-smoking females in China.

## Competing interests

The authors declare that they have no competing interests.

## Authors' contributions

ZY carried out the molecular epidemiological studies, participated in SNP detection and statistical analysis and drafted the manuscript. MS and ML participated in DNA extraction and SNP detection. XL and RM participated in sample collection and data acquisition. QH participated in the design and coordination and helped to draft the manuscript. BZ supervised the study, participated in its design and statistical analysis and reviewed the manuscript. All authors read and approved the final manuscript.
